# Investigation of the correlation between platelet antibodies and peripheral blood cytopenia in patients with hepatocellular carcinoma

**DOI:** 10.1038/s41598-024-60603-8

**Published:** 2024-04-27

**Authors:** Rui Han, Hui-Chan He, Wan-Ning Zhang, Ze-Tao Pan, Chen-Yang Feng, Ming-Jian Liang, Mei-Yi Wu, Wei Zhong, Wen-Qian Lin

**Affiliations:** 1 Laboratory of Oncology in South China, Department of Blood Transfusion, Guangdong Provincial Clinical Research Center for Cancer, Sun Yat-sen University Cancer Center, 651 Dongfeng Road East, Yuexiu District, Guangzhou, 510060 People’s Republic of China; 2 Department of Internal Medicine, Huangpu Hospital of Traditional Chinese Medicine, 3 Xieshan Road, Huangpu District, Guangzhou, 510700 People’s Republic of China

**Keywords:** Hepatic carcinoma, Peripheral blood cytopenia, Thrombocytopenia, Anemia, Platelet pharmacological antibody, Cancer, Immunology, Diseases, Medical research

## Abstract

The primary triggers that stimulate the body to generate platelet antibodies via immune mechanisms encompass events such as pregnancy, transplantation, and blood transfusion. Interestingly, our findings revealed that a subset of male patients with hepatocellular carcinoma (HCC), despite having no history of transplantation or blood transfusion, has shown positive results in platelet antibody screenings. This hints at the possibility that certain factors, potentially related to the tumor itself or its treatment, may affect antibody production. To delve the causes we initiated this study. We employed a case–control study approach to analyze potential influential factors leading to the positive results via univariate and multivariate regression analysis. We utilized Kendall’s tau-b correlation to examine the relationship between the strength of platelet antibodies and peripheral blood cytopenia. Antitumor medication emerged as an independent risk factor for positive results in HCC patients, and the strength of platelet antibodies positively correlated with the severity of anemia and thrombocytopenia. Without history of blood transfusion, transplantation, pregnancy, those HCC patients underwent recent tumor medication therapy are experiencing peripheral erythrocytopenia or thrombocytopenia, for them platelet antibody screenings holds potential clinical value for prevention and treatment of complications like drug-immune-related anemia and/or bleeding.

## Introduction

Platelet antibodies bear a significant correlation with various conditions such as fever nonhemolytic transfusion reactions, platelet transfusion failure, post-transfusion purpura, fetal and neonatal alloimmune thrombocytopenia, transfusion-related acute lung injury, as well as transplant rejection and survival^1–7^. The incidence of platelet antibody in both tumor patients and stem cell transplantation patients approximately ranges from 10 to 66%^8–10^. Blood transfusion, pregnancy, and transplantation are the principal immunological routes that instigate the production of platelet antibodies. Few studies have investigated whether certain pathways or factors intrinsic to the tumor itself or its treatment course could lead to positive platelet antibody screening results. Hence, this study focuses on patients with liver cancer, aiming to uncover other potential triggers influencing the production of platelet antibodies. Additionally, the correlation between these antibodies and peripheral blood cells is analyzed, aiming to provide references for the clinical prevention and treatment of anemia and bleeding complications in liver cancer patients.

## Materials and methods

### Ethics statement

The studies involving human participants were reviewed and approved by the Institutional Review Board of Sun Yat-sen University Cancer Center. And all experiments were performed in accordance with relevant guidelines and regulations. The patients/participants provided their written informed consent to participate in this study.

### Participants

This study involves patients with primary liver cancer who had platelet antibody screening conducted at the blood transfusion laboratory of Sun Yat-sen University Cancer Center between November 1, 2019, and March 31, 2023. All participating patients provided informed consent and met the inclusion criteria: they were between 18 and 79 years old, were conscious, and had been diagnosed with hepatocellular carcinoma by the pathology department of our center. The following conditions were grounds for exclusion: multiple primary tumors, human immunodeficiency virus infection, idiopathic thrombocytopenic purpura, systemic lupus erythematosus, rheumatoid arthritis, hyperthyroidism, sepsis, a history of transplantation, child–pugh grade C liver function, as well as a physical activity status (performance status) score of 3–4 points.

### Methods

**Figure 1:**
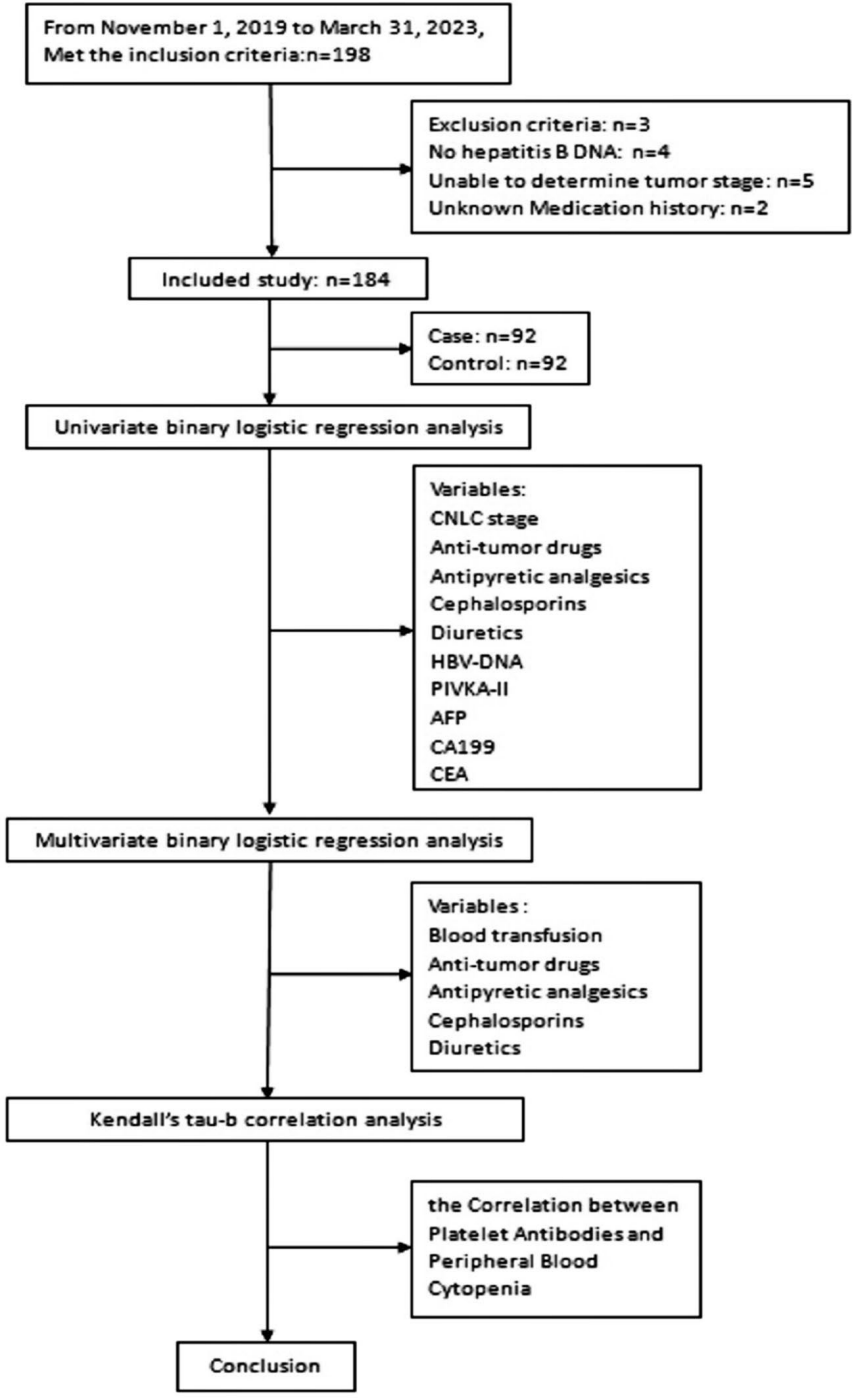
Flow diagram of study.

Patients who met the inclusion criteria and had positive results from the platelet antibody screening, based on time sequence, were included in the case group. The control group consisted of patients with negative results who were matched 1:1 to the case group based on the same sex and age within ± 3 years. Clinical data was gathered by reviewing electronic medical records or interviewing patients. The primary data includes: China liver cancer staging (CNLC), history of blood transfusions, and a three-month retrospective review of the patient's pharmaceutical history, particularly focusing on antitumor drugs, antipyretic analgesics, cephalosporins, and diuretics. Data results from platelet antibody screenings performed concurrently with other tests were gathered from patients. These tests included serum hepatitis B virus deoxyribonucleic acid (HBV-DNA), abnormal prothrombin induced by vitamin K absence-II (PIVKA-II), alpha-fetoprotein (AFP), carcinoembryonic antigen (CEA), and carbohydrate antigen 199 (CA199). Additionally, routine blood index measurements were collected, which encompassed the white blood cell count (WBC), neutrophil absolute value (NE#), total red blood cell count (RBC), hemoglobin concentration (HBG), hematocrit (HCT), and total platelet count (PLT). The final analysis included a total of 184 patients with comprehensive clinical data. First, a univariate analysis of all variables was conducted. Variables that displayed statistically significant differences in the univariate analysis, and that might have a significant impact on antibody screening, were subsequently subjected to a multivariate analysis. Finally, the correlation between the strength of platelet antibodies (categorized as negative, weakly positive, or positive) and the count of peripheral blood cells, the severity of anemia in liver cancer patients, and the level of thrombocytopenia were analyzed and evaluated (Fig. 1).

### Anemia degree classification

Based on the standard reference range for HBG in the clinical laboratory of our hospital (measured in g/L), which is 130–175 for males and 115–150 for females, anemia is categorized as follows: mild anemia is denoted by 90–130 for males and 90–115 for females. Moderate anemia is characterized by an HBG level of 60–90, while severe anemia is indicated by a range of 30–60. The above categories do not include caps. Very severe anemia is designated by an HBG level of less than 30. An HBG level greater than the upper normal limit is indicative of elevated hemoglobin concentration.

#### Classification of thrombocytopenia

Based on the standard reference value range of PLT count in our hospital's Clinical Laboratory, which is 100–350 (10E9/L), thrombocytopenia is classified into the following grades: Grade I ranges from 75 to 100, Grade II spans 50–75, and Grade III covers 25–50. These categories do not include any caps. Grade IV is classified as any count less than 25. Any PLT count greater than 350 is considered an elevated plate.

### Platelet antibody screening test

The test was executed in strict accordance with the standard operating procedure prescribed for the Dutch Sancun Platelet Antibody Screening Kit, utilizing the solid phase agglutination method^11,12^.

#### Experimental procedure

Firstly, the kit was allowed to reach room temperature, between 18 and 25 °C. Secondly, the concentrated wash liquid was diluted 25 times with distilled water to prepare the working solution. Furthermore, the concentrated wash liquid was also diluted with distilled water in a 1:24 ratio. Thirdly, a platelet suspension is prepared by reconstituting lyophilized platelets which are then used directly. Add 500 μL of normal saline to each branch, allow it to stand at room temperature for 5 min, and ensure thorough mixing. Finally, add 50 μL of platelet antibody screening cells to each reaction well. Gently shake for 10 s before centrifuging the platelets for 5 min. Afterwards, wash three times with washing solution. Carefully discard the washing solution, taking care not to apply too much force or pat. Swiftly add 100 μL of low-ionic strength solution to each reaction well, including the negative and positive control wells. Then add 50 μL of patient samples. After sealing each reaction well, gently shake the sealing film. Incubate the well in a 37 °C gas bath for 35 min. Discard the sealing film and remove the liquid from the plate well, wash the plate 5 times, again taking care not to apply too much force. Dry with absorbent paper and do not beat; add 50 μL of anti-human IgG. Then, add 50 μL of indicator red blood cells, ensure to mix the indicator red blood cells before adding to the solution. Gently shake and immediately centrifuge the platelets for 5 min.

#### Interpretation of results

**Figure 2:**
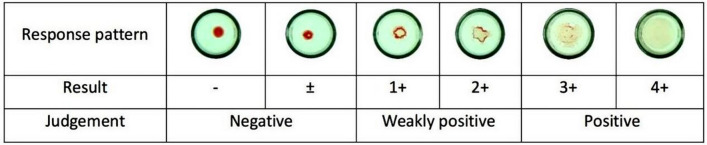
Platelet antibody detection response pattern.

For positive results: this suggests that the red blood cells are settled flat on the bottom surface of the reaction aperture. If the red blood cells are seen to be attached only to the base of some apertures, along with irregular protrusions around the cell’s outer edge, and if the attached area is larger than that of the negative control, this signifies a weakly positive result. This further indicates the presence of platelet antibodies in the patient’s serum or plasma. For negative results: this suggests that red blood cells assemble into an aggregation at the center of the reaction pore's base, or the area of red blood cell aggregation may exhibit a slight indentation, yet the aggregation area remains smooth. This implies that the patient’s serum or plasma does not contain any platelet antibodies. If the results appear dubious, or if the positive and/or negative controls fail to exhibit the expected outcomes, the test is repeated (Fig. 2).

### Statistical analysis

IBM SPSS Statistics software was utilized for all analyses. P < 0.05 was deemed statistically significant and all tests were two-sided. The normally distributed measurement data were analyzed using the independent sample t-test and represented as mean ($\overline{X}$ ± s), while the measurement data not adhering to a normal distribution were analyzed using a non-parametric rank sum test and represented as median. The statistical data are presented as cases and percentages (%) and were analyzed using the χ^2^ test. Multivariate logistic regression analysis was utilized for further evaluations. For correlation analysis, Kendall’s tau-b was employed.

## Results

### General data

In this study, data was collected from a total of 198 patients. This included 2 patients with multiple primary neoplasms, 1 patient with extremely poor liver function, 4 patients with missing HBV-DNA, 5 patients with missing CNLC, and 2 patients with an unknown medication history. After excluding certain patients, we ultimately included 184 patients, aged between 29 and 79 years, with primary liver cancer in this study. This cohort consisted of 138 males and 46 females, with 92 testing positive (case group) and 92 testing negative (control group). There was no significant statistical difference in terms of age, gender, and history of blood transfusion between the two groups (P > 0.05) (Table 1).

**Table 1:** Patient general informations.

General informations		Positive group (n = 92)	Negative group (n = 92)	X^2^/U	P*
Age (years)	Mean age	55.68	55.79	0.068	0.946
Gender (n, %)	Male	69, 75%	69, 75%	0	1
	Female	23, 25%	23, 25%		
Blood transfusion* (n, %)	Yes	12, 13%	6, 6.5%	2.217	0.137
	No	80, 87%	86, 93.5%		

*P > 0.05 was not deemed statistically significant.

### Univariate analysis

No significant differences were found in the CNLC, serum HBV-DNA, PIVKA-II, AFP, CEA, CA199, and the use of diuretics within a three-month period between the case and control groups (P > 0.05). However, significant variances were observed in the usage of antitumor drugs, antipyretic analgesics, and cephalosporin drugs within the same period in the two groups (P < 0.05) (Table 2).

**Table 2:** Univariate analysis.

Variables		Positive group (n = 92)	Negative group (n = 92)	X^2^/U	P
CNLC (n, %)	Ia, 1b	40, 43.5%	47, 51.1%	4.558	0.102
	IIa, IIb	7, 7.6%	13, 14.1%		
	IIIa, IIIb	45, 48.9%	32, 34.8%		
Antitumor drugs(n, %)	Yes	34, 37%	17, 18.5%	7.840	0.005*
	No	58, 63%	75, 81.5%		
Antipyretic analgesics (n, %)	Yes	34, 37%	21, 22.8%	4.383	0.036*
	No	58, 63%	71, 77.2%		
Cephalosporins (n, %)	Yes	31, 33.7%	17, 18.5%	5.525	0.019*
	No	61, 66.3%	75, 81.5%		
Diuretics (n, %)	Yes	27, 29.3%	24, 26.1%	0.244	0.621
	No	65, 70.7%	68, 73.9%		
HBV-DNA(n, %)	> 1000 COPY/ML	24, 26.1%	23, 25%	0.029	0.866
	< = 1000 COPY/ML	68, 73.9%	69, 75%		
PIVKA-II (mAU/mL)	Median	227	162	4168	0.859
AFP (ng/mL)	Median	22.10	93.63	3784	0.215
CA199 (U/mL)	Median	24.5	20.41	3916.5	0.382
CEA (ng/mL)	Median	2.94	2.65	3571	0.067

*P < 0.05 was deemed statistically significant.

### Multivariate binary logistic regression analysis

There was a heightened probability of positive platelet antibody screening observed in patients administered with antitumor drugs within the 3 months compared to those not given such medication (OR = 2.232, 95%CI: 1.016–4.904, P < 0.05). This implies that antitumor drugs independently contribute as a risk factor for positive platelet antibody screening in liver cancer patients (Table 3).

**Table 3:** Multivariate binary logistic regression analysis.

Variables	OR (95% CI)	P
Blood transfusion		
No	1.00	0.410
Yes	1.575 (0.535–4.636)	
Antitumor drugs		
No	1.00	0.046*
Yes	2.232 (1.016–4.904)	
Antipyretic analgesics		
No	1.00	0.267
Yes	1.755 (0.651–4.732)	
Cephalosporins		
No	1.00	0.160
Yes	1.774 (0.797–3.952)	
Diuretics		
No	1.00	0.126
Yes	0.467 (0.176–1.238)	

OR, odds ratio; CI, confidence interval.

*P < 0.05 was deemed statistically significant.

### Correlation analysis of platelet antibodies anvüd peripheral blood cells

**Figure 3:**
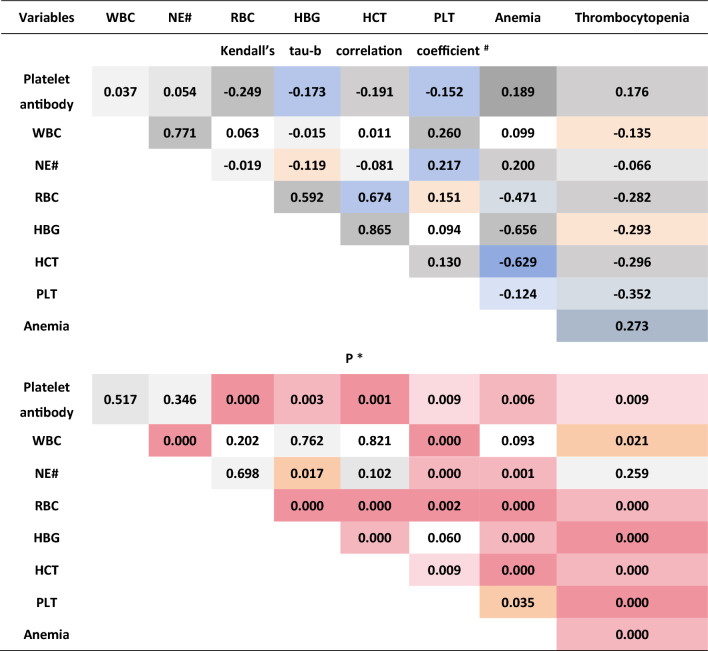
Kendall’s tau-b correlation analysis. ^#^Kendall’s tau-b > 0, showed positive correlation. Kendall’s tau-b < 0, showed negative correlation. *P < 0.05 was deemed statistically significant.

Kendall's tau-b correlation coefficient analysis was employed to evaluate the association between platelet antibodies and peripheral blood cells in HCC patients. The findings revealed no significant correlation between WBC, NE#, and the outcomes of platelet antibody screening (P > 0.05).The indices of RBC (Kendall's tau-b = − 0.249, P < 0.001), HBG (Kendall's tau-b = − 0.173, P < 0.01), HCT (Kendall's tau-b = − 0.191, P < 0.01), and PLT (Kendall's tau-b = − 0.151, P < 0.01) demonstrated a significant inverse correlation with the intensity of platelet antibody screening results. This suggests that as the antibody intensity increases from weak to strong, the aforementioned index values decrease correspondingly from high to low. Further analysis reveals a notable positive correlation between the intensity of platelet antibody screening results and the severity of anemia in liver cancer patients (Kendall’s tau-b = 0.189, P < 0.01), as well as the degree of platelet reduction (Kendall’s tau-b = 0.176, P < 0.01). This suggests that as the positive intensity of the platelet antibody screening increases, both the anemia and platelet reduction become more severe (Fig. 3).

## Discussion

The antigen system expressed on the surface of platelets is consisting of two main categories:human platelet antigen (HPA) which is found on the platelet membrane glycoprotein,non-specific platelet antigens which are common to platelets and other cells or tissues including the human leukocyte antigen (HLA) system, red blood cell groups ABH, Lewis,CD36, and others. In some cases (such as autoimmune diseases, pregnancy, transplantation and long-term blood transfusion, etc.), it can cause platelet antigen alloimmune response and produce corresponding antibodies,which are intricately linked to cellular immunity^13–15^. There are two types of platelet antibodies: one is antibodies against Class HLA-I antigens (PAIgG), and the other is platelet-specific antibodies against platelet membrane glycoprotein antigens (PBIgG). It is of great clinical significance to preliminarily understand whether patients have platelet-related autoimmune or alloimmune-related diseases through platelet antibody screening tests.

In this study, we found that the results of platelet antibody screening in patients with liver cancer were not associated with the tumor itself, but rather with the anti-tumor drugs. It was observed that the strength of platelet antibodies in liver cancer patients had a negative correlation with the count of peripheral blood cells, excluding white blood cells and neutrophils. Furthermore, a positive correlation was identified between the strength of platelet antibodies and the severity of anemia as well as the level of thrombocytopenia. The findings imply that whenever anemia or bleeding symptoms are observed post anticancer drug administration, it's critical not only to exclude the possibilities of acute and chronic blood loss, hypersplenism, malnutrition, tumor and bone marrow invasion, but also to diagnose the presence of hematological toxic reactions such as bone marrow suppression induced by anticancer drugs, and to identify any associated immune factors or drug antibodies.

To date, approximately 100 types of drugs, encompassing the aforementioned categories, have been reported in studies to cause alterations in the structure of platelet membranes, leading to the formation of neoantigens. These neoantigens stimulate the host to produce antibodies, subsequently forming a drug-platelet-antibody complex. This complex is cleared by the reticuloendothelial system, which results in thrombocytopenia in patients, and in some cases, leads to various forms of hematocytopenia^16–19^. Antibodies triggered by certain drugs may result in drug-dependent immune thrombocytopenia and drug-induced autoimmune thrombocytopenia. The alteration in the platelet membrane configuration elicited by the first category of drugs is usually reversible. Consequently, platelet levels typically rebound within several days following the cessation of the sensitizing drug. However, for the latter category, drug-induced autoantibodies may persist and continue to cause platelet destruction, even in the absence of the instigating drug^20,21^. Reports have surfaced of a complication involving a sharp and severe dip in platelet count in patients battling drug-induced thrombocytopenia (DITP). This phenomena is primarily observed in women undergoing treatment with platinum-based chemotherapy agents^16,22–24^. In rare circumstances of acute immune pancytopenia, one can witness oxaliplatin-induced thrombocytopenia. The acute stages of thrombocytopenia, hemolysis, and neutropenia are driven by the generation of oxaliplatin-dependent antibodies, which target platelets, erythrocytes, and neutrophils respectively. This has been reported in various literary sources^24–27^. Flow cytometry is capable of identifying oxaliplatin-dependent IgG antibodies in a patient’s serum, providing conclusive lab diagnosis^28–30^.

In this study, FOLFOX, a regimen consisting of fluorouracil, calcium folinate, and oxaliplatin, was prevalently utilized in therapies of liver cancer. This regimen may lead to hematological adverse reactions. Therefore, platelet antibody screening is of great significance between the end of the first antitumor drug treatment and the next administration. Those testing positive should be further investigated for the existence of drug antibodies akin to HPA or HLA-1 antibodies and their corresponding mechanisms of action. This would enable early intervention to prevent symptom aggravation and potential termination of tumor treatment. Further studies are still needed to confirm these observations.

The study presents with several limitations. Firstly, circumventing selective bias within our research subjects proves challenging. Secondly, obtaining comprehensive patient information data often encounters the hurdle of recall bias. Furthermore, it's not feasible for us to ascertain the antibody positive rate within the entire liver cancer cases. We anticipate future research with larger sample sizes and relevant randomized controlled trials to mitigate these issues.

## Conclusions

By utilizing regression models to minimize bias, it was determined that antitumor drugs independently contribute to positive platelet antibody screening results in primary liver cancer patients. Notably, the degree of platelet antibody was found to be in positive correlation with the severity of anemia and thrombocytopenia in hepatocellular carcinoma. For the prevention and management of immune disorders, neoplastic anemia, thrombocytopenia, and ineffective platelet transfusion occurrences during anti-tumor therapy, it is recommended that platelet antibody screening be performed promptly for patients undergoing treatment with combined anti-tumor drugs and peripheral blood cytopenia. Early detection and treatment of any arising issues are necessary to ensure the seamless progression of tumor treatment.

## Data Availability

The original contributions presented in this study are Included in the article/supplementary material, further inquiries can be directed to the corresponding author/s.

## Abbreviations

CA199: Carbohydrate antigen 199
CEA: Carcinoembryonic antigen
AFP: Alpha-fetoprotein
CI: Confidence interval
CNLC: China liver cancer staging
DITP: Drug-induced thrombocytopenia
HBG: Hemoglobin concentration
HBV-DNA: Hepatitis B Virus deoxyribonucleic acid
HCC: Hepatocellular carcinoma
HCT: Hematocrit
HLA: Human leukocyte antigen
HPA: Human platelet antigen
NE#: Neutrophil absolute value
OR: Odds ratio
PIVKA-II: Abnormal prothrombin induced by vitamin K absence-II
PLT: Platelet count
RBC: Red blood cell count
WBC: White blood cell count
